# Nutritional care of medical inpatients: a health technology assessment

**DOI:** 10.1186/1472-6963-6-7

**Published:** 2006-02-02

**Authors:** Karin O Lassen, Jens Olsen, Edvin Grinderslev, Filip Kruse, Merete Bjerrum

**Affiliations:** 1 Health Technology Assessment Unit, University Hospital of Aarhus, Aarhus, Denmark; 2 Centre for Applied Health Services Research and Technology Assessment, CAST, University of Southern Denmark, Odense, Denmark; 3 Centre for Alternative Social Analyses, CASA, Copenhagen, Denmark; 4 State and University Library, University of Aarhus, Aarhus, Denmark; 5 Institute of Public Health, Department of Nursing Science, Faculty of Health Sciences, University of Aarhus, Aarhus, Denmark

## Abstract

**Background:**

The inspiration for the present assessment of the nutritional care of medical patients is puzzlement about the divide that exists between the theoretical knowledge about the importance of the diet for ill persons, and the common failure to incorporate nutritional aspects in the treatment and care of the patients. The purpose is to clarify existing problems in the nutritional care of Danish medical inpatients, to elucidate how the nutritional care for these inpatients can be improved, and to analyse the costs of this improvement.

**Methods:**

Qualitative and quantitative methods are deployed to outline how nutritional care of medical inpatients is performed at three Danish hospitals. The practices observed are compared with official recommendations for nutritional care of inpatients. Factors extraneous and counterproductive to optimal nutritional care are identified from the perspectives of patients and professional staff. A review of the literature illustrates the potential for optimal nutritional care. A health economic analysis is performed to elucidate the savings potential of improved nutritional care.

**Results:**

The prospects for improvements in nutritional care are ameliorated if hospital management clearly identifies nutritional care as a priority area, and enjoys access to management tools for quality assurance. The prospects are also improved if a committed professional at the ward has the necessary time resources to perform nutritional care in practice, and if the care staff can requisition patient meals rich in nutrients 24 hours a day. At the kitchen production level prospects benefit from a facilitator contact between care and kitchen staff, and if the kitchen staff controls the whole food path from the kitchen to the patient. At the patient level, prospects are improved if patients receive information about the choice of food and drink, and have a better nutrition dialogue with the care staff. Better nutritional care of medical patients in Denmark is estimated to hold a cost savings potential reaching approximately USD 22 million.

**Conclusion:**

Every hospital and every bed ward has its strengths and weaknesses, but none of the participating bed wards fully satisfy nutritional care success criteria. All organisational levels have a significant potential for improvements of nutritional care of medical inpatients.

## Background

Many patients' food intake is insufficient during hospitalisation, although their protein and energy intake can be increased significantly by optimising the nutritional care using available hospital food [1]. The focus on elderly medical patients' nutritional care is rooted in surprise over the distance between recognised theoretical knowledge of how important nutrition is to the patient, and the frequent failure to integrate nutritional care in the overall treatment and care for patients [2-4]. Among elderly inpatients, 20–50 percentage are undernourished [5]. Other studies within this field also demonstrate that it is possible to satisfy the patients' nutritional requirements [6-10]. The Official Danish Food Recommendations for Institutions transform the clinical knowledge of patients' nutritional needs into guidelines for the nutritional care in hospitals [11]. In spite of these official recommendations, which are intended as a guide and the political focus on the issue, practical nutritional care is still not given a high priority, and it is difficult within the organisation of the hospitals to optimize the nutritional care [3,10,12,13]. Elderly medical patients constitute the largest single group of hospitalised patients in Denmark, and a large group of patients is accordingly subject to a higher risk of complications, longer convalescence, and longer hospital stays [14].

Danish studies of patients' nutrition often address issues concerning running of the production kitchens or the clinical importance of nutrition at the expense of an overall perspective on nutritional care per se. Nutritional care is defined as the basic duty to provide adequate and appropriate food and drinks to the patients [15].

Subjecting nutritional care to a health technology assessment (HTA) allow nutritional care to be seen in a holistic perspective, and will at the same time afford greater insight into the nature of those factors that determine whether medical patients are receiving optimal nutritional care, and hence how the risk of under nourishment may be minimised. From official side a cost benefit analysis including all somatic hospitalisations was performed in 1997 [16]. Since this analysis do not specifically examine the economic implication of improved nutritional care of medical inpatients we found it important to perform a more detailed analysis for this specific patient group.

The aim of this study is threefold: 1) To identify, describe and analyse problems in the nutritional care of elderly medical inpatients. Nutritional care is defined as the basic duty to provide adequate and appropriate food and drinks to the patients. 2) On this basis to consider changes in the nutritional care necessary to ensure the satisfaction of the patients' nutritional requirements. 3) In a health economic analysis to describe and analyse the costs, and effects of an improved nutritional care. This aim is in close accordance to the methodology of the HTA, which facilitates a comparison of diverse elements of the nutritional care obtained by different analytical approaches.

## Methods

### Design of study

In order to provide a systematic and comprehensive foundation for the present HTA, data have been collected on four main aspects: the technology, the organisation, the patient, and the economic aspects [17]. The Official Danish Food Recommendations for Institutions include guidelines for the nutritional care in hospitals [11]. These recommendations describe the distribution of tasks and responsibilities among the different professional groups, the composition of the food, conditions for the serving of the patients' meals, and information about the food served. These recommendations are generally the basis of nutritional initiatives taken in the national hospitals. For this reason, the recommendations have been chosen as the analytical framework, and have for this purpose been transformed into six criteria of success:

1. The diet is an important element in the overall care irrespective of the duration of hospital admission

2. The nursing staff plan the diet individually in accordance with each patient's requirements and wishes

3. The patients nutritional status is monitored during admission, and the ward staff act in accordance herewith

4. There is a clear division of responsibility and tasks relating to nutritional care

5. The food is appealing and nutritionally appropriate

6. The nursing staff ensure that the patient's mealtime are as pleasant as possible

These six criteria of success are the turning-point in the HTA. In order to elucidate how to improve the nutritional care for elderly medical inpatients, we examined how nutritional care is performed at medical wards in three Danish hospitals. The nutritional care is defined as the duty, which provide adequate and appropriate food and drinks to the patients. The criteria for a medical ward to be included in the study are as follows:

1. The hospital's production kitchen deliver hot food to the bed wards, where the meals are portioned out. This criteria ensures that the fundamental conditions for the food service in the participating bed wards are as comparable as possible.

2. The staff in the medical ward is experienced in implementing the official recommendations for the nutritional care – e.g. through nutrition activists and management's initiatives.

Three hospitals participated in the study; a university hospital (A), a local hospital (B), and a hospital in the Copenhagen Hospital Corporation (C). The practices in the nutritional care at the medical wards at the three hospitals are compared with the six success criteria for nutritional care to trace factors that promote or inhibit optimal nutritional care from the perspectives of the patients and the professional and managerial stakeholders.

### The technology

The technology was formulated as early as in 1860 by Florence Nightingale, who wrote "I would say to the nurse, have a rule of thought about your patient's diet, consider, remember how much he has had and how much he ought to have today" [18]. In this HTA, the technology is defined as "the nutritional care that ensures that the patient's nutritional requirements are satisfied to the greatest extent possible". The significance of optimal nutritional care during illness, and possible means to enhance the nutritional intake of patients who eat very little, is described by means of a systematic literature search.

### The organisation

The organisation is the key element of the HTA as the organisational analysis provides insight into how nutritional care is currently practised by the professional groups in three hospital medical wards. The professional groups are interviewed in focus groups or individual interviews about their responsibilities, their work regarding nutritional care, and their experience in nutritional care as practiced [19]. These groups include nursing staff, kitchen staff, key figure for nutritional care, clinical dieticians, catering officers, and representatives of management at the hospitals (head matrons, doctors in management positions, and representative of the hospital management). The turning-point in the interviews concerns how the six criteria of success are complied with, and which factors inhibit or promote the fulfilment of the criteria. As background for the structured interviews, information is collected on measures implemented in the nutritional care area by the hospital owners and management as well as the running of the hospital wards and the production kitchens [20]. The interviews are tape-recorded with the permission of the informants. The transcribed interviews are analysed as a text, and through the organisational analysis, factors are identified that promote or inhibit optimal nutritional care [19].

### The patients

In order to elucidate the patients' experience of and views on nutritional care (defined as nursing providing adequate and appropriate food and drinks to the patients) related to the six success criteria, a questionnaire is prepared for use in a structured interview with 25 medical patients from each participating medical ward unit (in total 75 patients). The questions are designed to focus on issues assumed to be related to the patients nutritional care, and to reflect their experience with the food services from a consumer's point of view [21-23]. The patients are interviewed separately in a quiet place to allow them to answer questions and give comments undisturbed [24]. Questionnaire comments made by the patients yield additional information that serve to increase response validity [25]. Based on the patients' answers and open comments, factors are identified that from the patients' perspective promote or inhibit fulfilment of the six success criteria for nutritional care. The included patients have been hospitalised for at least five days, do not receive a prescribed diet or nutritional support, and are able to communicate. All the patients have prior experiences as users of the nutritional care, and all questions are related to that subject. Participation is voluntary.

### The economy

**Figure 1:**
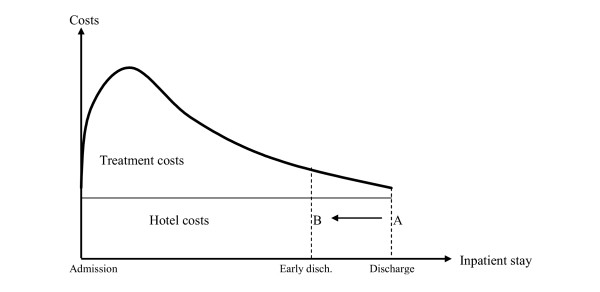
**Correlation between duration and costs of admission. **The figure shows that an improved nutritional care entails a (marginal) reduction in the hospitalisation period at the end of the period. Hotel costs include costs related to food, care, linen, cleaning, buildings and the like and the daily hotel cost is calculated as the bed day charge used in the DRG system for disbursement of hospital days in excess of the normal hospital admission period. (From Drummond MF, O'Brien B, Stoddart GL, Torrance GW. *Methods for the economic evaluation of health care programmes*. 1997: 63.)

The international literature describes costs and effects of improved nutritional care. These results are supplemented with data concerning the activity levels (that is, number of admissions) at the participating hospital units and hence related to the Danish context to provide a background for estimation of the costs and effects of improved nutritional care. The cost analysis focuses on the potential for saving hospital days, and it is assumed that one hospital day saved corresponds to the cost of staying for one day in a hospital (figure 1). The assumption is that improved nutritional care entails a (marginal) reduction in the hospitalisation period at the end of the period. Hotel costs include costs related to food, care, linen, cleaning, buildings and the like, and the daily hotel cost is calculated as the bed day charge used in the Diagnosis-Related Groups (DRG) system for disbursement of hospital days in excess of the normal hospital admission period [26]. The DRG system determine the actual costs of the hospital treatment of individual patients, and calculate the average treatment costs for the patients in the individual patient group. It is estimated that this charge reasonably precisely reflects the costs of hospital days at the end of a period of hospitalisation. The charge is USD 224 per day [26]. This calculation only includes hospitalisation courses for patients above the age of 59 years, who are hospitalised for at least 7 days. In the baseline it is assumed that 35 percentage of these patients are undernourished (the average of 20 and 50 percentage [5]) and a potential average of 3.4 days reduction in hospital stay is also assumed – that is a reduction in conformity with the Cochrane review [14] (accessed first quarter 2003). The cost calculations rest on a number of assumptions, and a simple one-way sensitivity analysis is therefore performed with variation of computational premises.

## Results

### The technology

Diseased individuals, who have been exposed to bodily stress, have increased energy and nutritional requirements compared with healthy individuals, because the body's exchange of nutrients rises in response to disease [1]. If the patient does not receive "the care that ascertains that the patient's nutritional requirements are satisfied to the greatest extent possible", (i.e. if the technology is not used optimally), muscle tissue will be consumed, including heart and respiratory muscle, and the immune defence will be weakened. The patient will tire and become difficult to mobilise, which entails a risk of complications like pressure ulcers, phlebitis, and infections and hence an increased risk of longer hospitalisation [1,14]. Studies have shown that the patients' nutritional intake (special energy and protein) can be optimised by serving small meals, where the concentration of nutrients is increased, but the volume of the food is unchanged [7,10]. Similarly, the patients' nutritional intake is increased by serving frequent between-meals [9]. If the patients have the opportunity to choose tempting meals that are also rich in nutrients as a supplement to the standard hospital diet, their nutritional intake can even be increased [8]. A Danish project elucidates the importance of hiring an nutrition assistant (i.e. a social and health assistant who has received supplementary education and training in nutritional care), whose job is to ensure that nibblers' nutritional requirements are satisfied to the greatest extent possible. This job increases the patients' nutritional intake, minimises the amount of food wasted, and increases the other care staff's focus on nutritional care [6].

### The organisation

In total 60 representative from the professional groups are interviewed in one of the 22 focus groups or individual interviews performed. These groups include 28 from the nursing staff, two kitchen staff, three staff assuming key positions in relation to nutrition, four clinical dieticians, ten catering officers/assistants and 13 representatives of management (four head matrons/head of the production kitchen, three nursing officers, three doctors occupying leading positions and three representative of the hospital management). At Hospital A 13 staff members are interviewed, at Hospital B 23 staff members, and at Hospital C 24 staff members, respectively. The organisational analysis of the participating hospitals and the bed wards shows that hospital owners, hospital management, and ward staff have different perspectives on nutritional care. The individual hospital and the individual bed section have their own particular strengths and weaknesses, but neither of the participating bed sections fully satisfied the six success criteria for nutritional care. In one hospital, management has not clearly indicated that the nutrition is an important part of the treatment. At two bed wards the care staff do not compose the menu according to patients' needs and wishes. Only one of the participating departments monitors the patients' nutritional status during hospitalisation. Only one of the participating departments clearly identify who is responsible for the actual performance of nutritional care, etc. Elements central to the achievement of optimal nutritional care are identified as factors that promote or inhibit optimal nutritional care from the perspective of the hospital management, the ward, and the production kitchen. The results are summarised in table 1.

At all the three hospitals the food is prepared centrally in the production kitchen, and is transported to the bed wards by food trolleys. At Hospital A and Hospital C the transportation is carried out by hospital porters. Hence, the kitchen staff is neither involved nor has influence on how the food is served at the bed wards. At hospital B this has been changed 15 years ago, so now the kitchen staff takes the food trolleys to the bed wards and prepare for the serving and the nursing staff serves the food. The staff in the production kitchen at Hospital B finds that frequent and positive contact with the care staff and patients at the bed wards is productive:

"It has increased the quality of the food that we stand face-to-face with the patients, who are going to eat the food. Before, it was easier to send off a meat roll that had been fried a little too much and then just close the food trolley and think 'we will probably hear nothing'. Today, it is not particularly nice to be standing over there and hear when the patients comment on the food."

Head matron, Hospital B

Hence, the staff in the kitchen gets the opportunity to constantly adjust and adapt their production to the actual needs for food and beverages of the patients. At the same time, the skilled kitchen staff gets the opportunity to follow their products all the way to the patient:

"It matters more to us in the kitchens that the food is appealing than it maybe does to the care staff, because this is not their area of competence. They don't think much about this. But it is the final result of the work we have been doing."

Head matron, Hospital B

The greater visibility of the kitchen staff in the bed wards increases the knowledge and the respect for the kitchen staff's jobs and professional competences:

"Our kitchen has always wanted to be at the forefront of developments and we have always appreciated the importance of the food. The kitchen wants to do whatever they can for the patients."

Nurse, Hospital B

Inversely, it is counterproductive to optimal nutritional care if no formalised, positive contact is established between the production kitchen and the bed wards. The production kitchens at Hospital A and Hospital C receive no feedback on the production of food and beverages delivered to the bed wards. The skilled staff in the kitchen accordingly does not know when and if the patients receive the different offers of food and beverages from the production kitchen:

"The food is not taken seriously. There is much information, and then it may not be those [at the wards], who are really serving the food who get the information. You sometimes think that you are really doing all you can – sending notes to one, then the other, and in spite of that they can call you – and nobody has seen the note!"

Assistant matron, Hospital A

When the kitchen staff has no say in how their products are served for the patients, it is counterproductive to optimize the food service. If the kitchen staff is not at any time organisationally visible, the care staff and department management have no opportunity of understanding and utilising this job function and staff competences. This represents a problem because the patients shall then not come to benefit from the kitchen staff's knowledge.

From the bed wards' perspective, nutritional care is furthered by the presence of staff that takes a particular interest in nutritional care like nutrition activists and staff assuming key positions in relation to nutrition. Hospital C has a strong focus on prevention, and has given nutritional care a high priority throughout the organisation. This has been accomplished through nutritional education to key staff members, education to all newly employed, and fixed standards for the nutritional care:

"Nutritional care is now integrated in our daily work, and social and health care assistants are now often the key personnel in this respect. And they are really good at this, which is one of their key competences. It is the right field in which to give them much responsibility and they take on that responsibility very well – most of them anyway."

Clinical head nurse, Hospital C

Nutritional care is furthered if the care staff can requisition between-meals 24-hours-a-day to be served alongside the five fixed meals, which are served from 8 a.m. to 8 p.m. Interdisciplinary guidelines for the treatment and care for specific patient groups (see e.g. [27]) often contain a description of nutritional care for these patients, and such guidelines can be instrumental in increasing the care staff's focus on nutritional care. However, our data generally show that the care staff at the wards lacks time to perform nutritional care. Such lack of time may cause this area to be under prioritised:

"Well, sometimes I do feel a pang of bad conscience. Because I do know that this is something I don't have time to do. It could be an ideal situation, but I don't think it is, because there is no time to do what should be done."

Social and health assistant, Hospital C

Nutritional care in the bed ward is often a collectively defined area of responsibility, but experience shows, however, that very few members of the care team take on this responsibility:

"If we did not have an assistant nurse like her – if she did not take responsibility, things would not work. We have no system, no control, and no plans."

Medical consultant, Hospital B

Often care staff focusing on the patients' nutrition does not have access to dietary supplements rich in energy and protein that they can give to the patients in between the fixed meals. According to the informant the staff ought to be able to serve such supplements to the patients' prescribed diet. Related to the responsibility for nutritional care another problem is that the doctors are responsible for evaluating the patients' nutritional status, and for securing that the care staff focuses on nutritional, but doctors only rarely demonstrate their nutritional commitment:

"Pro forma it should actually be the doctor who did the nutritional assessment when a patient was admitted. But, I think we have discussed that, this is not how it is in practice. As I see it, it is we who make these analyses. "

Nurse, Hospital B

At Hospital C, a precise delimitation of each professional group's area of responsibility has not meant that doctors take on responsibility for the nutrition:

"When the central guideline governing accreditation was introduced, it was clearly spelled out that it was the doctor's responsibility, when monitoring and dispensing and everything was done. We quickly found out that this was a bad idea, because nothing was done! And they do not focus on this now, and I think they will never admit that they never will."

Clinical head nurse, Hospital C

The clinical dieticians, who plan nutritional therapies for nibblers and patients whose nutritional status is threatened, often experience that their advice is ignored:

"When you visit the wards and plan a nutritional therapy, you agree that the staff should monitor the patient's weight and eating. When that is not done, well then it really annoys me."

Clinical dietician, Hospital C

A factor running counter to nutritional care optimisation is also that the clinical dieticians often are not involved in the nutritional care at the bed wards:

"I think, that we have a higher level of professionalism to offer. If you consider how the care staff uses our resources, well then you could often have implemented nutritional care at a higher level, i.e. at the level of nutritional support provided by the clinical dietician. And we lack coordination in this house; all bed sections do not have to have a group or persons or some nurses who invent things all over again when it comes to nutritional care. There is no coordination in this house."

Clinical dietician, Hospital A

From a hospital management perspective, nutrition can be improved if management clearly signals that nutritional care is important to hospitalised patients, and if it is clearly communicated which groups or staff are responsible for which areas:

"To me, it gives little sense to say that throughout the entire system, the doctor must make sure that the patient's dietary plan is optimal. We know that this is not part of what they work with; it would make sense to delegate this area of competence to a nurse or a social and health assistant. But focusing on the therapeutic role of nutrition is part of the medical profession, and we recognise that. To me, that might well be the basis of it all, but it should also be said that doctors can delegate tasks. So we have done a lot to integrate nutrition in the overall health care, knowing, of course, that it is an interdisciplinary area."

Representative of the hospital management, Hospital C

On top of these clear statements, it is argued that management should have access to management tools to ensure quality nutritional care, and that it shall continuously monitor that nutritional care is, indeed, performed:

"We greatly benefit from the ongoing accreditation process. We are presented with a number of standards that we must live up to and they must be implemented. I think we would have had these guidelines anyway, but the accreditation process has been a superb management tool, because somebody arrived, measured and evaluated our working processes, and we are trying to live up to these standards. We have committed ourselves to consider our results, how far people have come, and to follow up on these observations. And that is a good management tool."

Representative of hospital management at Hospital C

Nutritional guidelines are rarely implemented without active effort, and nutritional optimisation is therefore furthered by the availability of organisational resources for follow-up and support of these efforts. Inversely, absence of clear signals hamper the work throughout the hospital organisation, in particular if hospital management fails to clearly signal the therapeutic importance of nutritional care. And hospital management will in these cases rarely be motivated to check that nutritional care is performed at departmental levels:

"We do not have an actual evaluation of how nutritional care is being performed – we have not done this. And we actually should do this. But the thought probably has not occurred to us that there can be departments where these practices are not followed."

Representative of hospital management, Hospital B

### The patients

From each medical wards at the three hospitals 25 patients are interviewed (37 women and 38 men). The average age is 75 years old (minimum 40 years old, maximum 97 years old). On average the patients have been hospitalised for 16 days and nights when they are interviewed. The patients' answers and their comments to the questionnaires show that nutritional care is performed in different ways at different bed sections. But in general there is room for improvement in the nutritional care, in particular for offering the patients a care geared to their particular needs. In table 2 factors that promote or inhibit optimal nutritional care from the perspective of the patient are summarised. At the patient level, better nutritional care can be obtained if the patients are given a genuine opportunity to choose between diets. It will also aid nutritional care if the care staff tell the patients about the selection of food and beverages available from the kitchen, and if the care staff then order and serve the patients these meals. Nutritional care will, of course, also benefit if the presentation of the meals is attractive and is adapted to the individual patient's needs in terms of size and consistency. Patients do not always experience that this is the case.

*"When you are hospitalised, the meal is supposed to be the best time of the day – but I really don't know*.......... *"*

Patient, Hospital C

According to the interviews, the patients find that it is counterproductive when they are not given an opportunity to choose a menu satisfying their preferences and ability to chew:

"I eat what is served – if I do not like it, I simply don't eat it"

Patient at Hospital A

The fixed meals are served within a 12-hours-period, and it is consequently counterproductive to optimal nutritional care if the patients are not offered between-meals rich in energy and nutrients during the remaining 12 hours (i.e. from 8 p.m. to 8 a.m.), but only have access to water, juice, etc. which do not contribute to the nutritional intake:

"They always serve water. They do not ask what you would like to have – you can fetch juice yourself, but it is not easy if you are in a wheelchair"

Patient, Hospital A

The production kitchens offer a range of dishes and beverages rich in energy and protein. However, it is counterproductive to optimal nutritional care when the patients are not informed about these offers, and do not have the opportunity to have these meals, and/or when the dialogue between the patient and the care staff does not properly address the individual patient's dietary needs and preferences. However, often these patients do not express themselves clearly, saying that *"we cannot be too demanding. That is more for the young ones. When you are in a place like this, you have to adapt to the situation – you do get home again, after all"*.

Failure to follow the patients' nutritional status during the admission, and ignorance of the patient's own observations about into his or her weight loss, are also counterproductive to efforts to improve the nutritional care. Many patients have a clear feeling, whether they are loosing weight, and they know that food and beverages are vital:

"You have to eat and drink – otherwise you die"

Patient, Hospital C

### The economy

A literature review identifies a number of studies showing that improved (optimal) nutritional care significantly reduces hospitalisation [14,29]. Hence, a Cochrane review (accessed first quarter 2003) finds the average hospital admission time to be significantly shorter among patients who have received optimal nutritional care -3.4 days (95 percentage confidence intervals: -0.69 to -6.12 days) [14]. In the Cochrane review the patients' average age is 65 years or more; all patient groups are included except elderly patients in critical care and cancer patients, and nutritional intervention consists of increased intake of protein and energy in food and beverages. So, improved nutritional care will result in a (marginal) reduction in the duration of the inpatient stay. In addition, other studies report a significantly increased proportion of complications among undernourished patients [1,30-32]. Some studies report that improved/optimal nutritional care can reduce mortality [14,30,33]. One study reports a tendency to higher readmission among undernourished patients [28]. Finally, a number of studies suggest that functional levels are heightened by improved/optimal nutritional care [14].

Table 3 shows the number of hospitalised patients in 2001 and 2002 within the participating departments, and the fraction of patients aged 60 years or more who are hospitalised for more than seven days. Assuming that 35 percentage (the average of 20 and 50 percentage) of these patients are undernourished, and that the nutritional care is improved so that the average duration of their hospitalisation is reduced by 3.4 days in conformity with the estimates presented in the Cochrane Review [14] (accessed first quarter 2003), the annual gain in terms of bed days saved will reach 296 days (USD 66,412), 524 days (USD 117,355) and 441 days (USD 98,952) at the three participating departments (table 3).

Based on the organisational model, in which an nutrition assistant is in charge of nutritional care for nibblers, the costs saved are reduced to USD 26,181, USD 36,893 and USD 18,490, respectively (table 3). That is, the costs of employing one or two nutrition assistant (depending on the size of the department) are subtracted from the original estimated gain. The reason for using this model of organisation, is the experience from Bispebjerg Hospital, Copenhagen, Denmark. Here the employment of a specially trained nutrition assistant is solely in charge of the nutritional care for nibblers to improve their nourishment [6].

Extrapolation of the cost analysis to embrace all departments of internal medicine in Denmark yields the following scenario (table 4): The year 2001 has a total of 81,705 admissions in medical wards in Denmark of patients aged 60 years or older for periods of at least 7 days. Assuming that 35 percentage of these patients are under-nourished yields an admission figure of under-nourished patients of 28,597. Computations founded on assumptions of this kind always imply a certain level of risk. Table 5 therefore shows different estimates of reductions in inpatient stays calculated as annual cost reductions (sensitivity analysis), if the assumptions are included as variables. In Denmark, the approximated overall cost reduction potential amounts to USD 22 million based on a reduction in the number of bed days of 3.4 days for 35 percentage of the medical patients above 59 years of age who are admitted to hospital for at least 7 days. As stated, the savings potential varies with the assumptions used.

## Discussion

The qualitative data from the organisational analysis shows that the six success criteria, specified according to Danish public recommendations, are not fully met in the three participating bed sections. This result is supported by the results from other studies which conclude that there are ample room for improving the extent to which hospital food satisfies patients' protein and energy requirements (see e.g. [3,13,22]). Official dietary recommendations for hospitalised patients are consultative, and the individual actors, the hospital owners, the hospital management, the heads of departments, and the care staff, act very differently in terms of their priorities and work with nutritional care. The analysis shows that there is considerable potential for optimising nutritional care – even to a substantial degree – in all three hospitals.

As discussed in the section on technology, a number of opportunities are available for optimising the nutritional care for nibblers. Feasible opportunities include serving frequent, small meals rich in nutrients. The question remains, however, who will perform this work? Hiring an nutrition assistant has been shown to increase surgical nibblers' nutritional intake [6]. The positive results of this way of organisation are utilised because many of the problems in the nutritional care of nibblers are assumed to be comparable for medical and surgical inpatients. Obviously, an organisational model, in which a professional is hired exclusively to take care of tasks related to nutrition at the bed ward, meets four of the criteria required to raise nutritional standards at departmental levels: the nutrition assistant's time is devoted to providing individual nutritional care to nibblers; she has intimate knowledge and takes an interest in nutrition; she has general knowledge of the food range offered by the production kitchen with which she is in close contact. The patient will experience a range of opportunities, because she draws on the entire food repertoire available from the production kitchen, makes new suggestions in connection with the meals and between-meals offered by the production kitchen, and she produces small meals and pastry herself in the bed ward kitchenette.

This organisational model eliminates counterproductive factors like lack of time and responsibility evasion when the care group is going to decide who should perform the tasks, e.g. to persuade nibblers to eat. There will be a clear separation of jobs between the nutrition assistant and other staff. This cooperation model assumes that the staff understand the importance of the nutrition assistant's job, which may be achieved through education and training of other staff at the bed ward [6]. This model will also secure a more efficient use of resources in nutritional care, because the patients' nutritional requirements will be better met, and because the amount of food wasted can be significantly reduced [34]. The physical framework required by an nutrition assistant is a desk (2 × 1/2) m^2^, a dinner trolley of the same size, an oven, cupboard space for groceries and space for cooling and freezing. The model only moderately affects the physical framework within the bed wards, and it is estimated that the majority of the medical wards at Danish hospitals will be able to introduce the model.

Several aspects of this organisational model are also confirmed by the patient interviews in this project. The patients believe that there is room for optimising their nutritional care, and for their nutritional requirements to be met to a higher extent. Central to this effort is more information about the dietary services, improved dialogue with the staff/care providers based on the patient's needs and preferences, and the availability and offering of food 24-hours-a-day; in short, the possibility of having an individualised diet.

Hence, this study identifies several factors that either facilitate or hamper efforts to optimise nutritional care defined as nursing providing adequate and appropriate food and drinks to the patients within medical bed wards. Either by strengthening or eliminating these factors, the cleft between theory and practice can be narrowed, which reduces the number of undernourished patients in Danish hospitals. Such optimisation lead, among others, to a decline in the average duration of hospitalisation and a reduction in bed occupancy days at the departments of medicine of an estimated 6.7 percentage. Such a reduction helps ease the future pressure on the medical bed wards that follows in the wake of the ageing of the population in the years to come. Real cost reductions will only accrue if hospital bed days saved lead to a reduction in the number of beds and subsequently reduced staffing and reductions in associated costs. However, all other things equal, it will be possible to translate the cost saving potential into a reduction in the bed occupancy rate in departments of medicine where the current bed occupancy rate reaches 98–99 percentage, which should be compared with an optimal rate of less than 85–90 percentage [35].

The results from the cost analysis rests on the assumptions that the results from the Cochrane review can applied to a Danish setting of medical patients. This application can only be done with caution, and sensitivity analyses are necessary to test the robustness of the results. Compared to the cost benefit analysis carried out in [16] the present analysis seems more detailed, since the calculations only were performed for a specific patient group (medical inpatients above the age of 59 years, hospitalised for at least 7 days) and since fairly comprehensive sensitivity analyses were performed. The analysis in [16] assumed an average savings potential on 4 days per patient corresponding to a savings potential on USD 77 million (1997 price level). This is higher, among others because of a larger number of patients included in the analysis. The Cochrane review finds the average hospital admission time to be significantly shorter among patients who have received optimal nutritional care (-3.4 days), but this result includes surgical as well as medical patients, and even though the studies with surgical patients represented, a majority of the medical patients constituted 64 percentage of all the patients [14] (accessed first quarter 2003).

No studies have reported results of the effects of improved care on the general health condition. Nor have any studies sought to estimate gains in life expectancy as a result of improved nutritional care. The Cochrane Review reports that the scarcity of data and the brevity of most studies make it impossible to demonstrate any change in life quality [14]. The lack of relevant outcome measures implies that a measure like reduction in the duration of hospitalisation becomes a surrogacy for clinical outcome measures and health status. However, a reduction in hospital bed days due to improved nutritional care is associated with health status; when a patient obtains an earlier discharge, we can expect, all other things being equal, that the patient recovers more quickly and hence obtains a gain in the form of a better health status [14].

The present study includes three hospitals, but neither hospitals, professional groups nor patients can be assumed to be representative on a national level. The nutritional care, too is carried out in different ways e.g. variation in management of the kitchen, composition of menus, and the commitment to nutrition displayed by care staff and managements are all part of the larger picture. As hospitals work differently in the field of nutrition, no standard answer can be given as to how nutritional care shall be optimised. But still for the majority of Danish hospitals the nutritional care consists of a long series of combined work operations and processes which run from the production kitchen and up to the patient. In this respect there are no significant differences as to how the nutritional care is located within the organisation, and which priority it is given. The organisational position and prioritisation of nutritional care are, therefore, basically assumed to be comparable. The nature of the problems that are related to insufficient nutrition of hospitalised medical patients are accordingly assumed to be of a similar nature in Danish hospitals and in some European hospitals [12]. The individual hospital can hence benefit from an assessment of individual factors facilitating or inhibiting nutritional optimisation within their respective organisations.

The topicality of this issue is reinforced by the fact that Denmark has now adopted the European Council's recent resolution concerning food and nutritional care in hospitals [15]. This specifies that a well-functioning meal service and individual nutritional care are fundamental human rights for hospitalised patients.

## Conclusion

Official nutritional recommendations for hospitalised patients are not observed in full by the participating hospitals, and the health technology assessment (HTA) survey discloses a number of factors or situations that either facilitate or hamper optimisation of nutritional care. The individual organisation has a considerably potential for optimising the nutritional care. This study concludes that from the perspective of patient ethics and resource optimisation in the treatment and care at Danish hospitals, it is of central importance to give priority to sufficient nutrition of hospitalised medical patients. Overall, long-term planning and implementation of optimal nutritional care in a viable organisation offer a qualified perspective.

## Competing interests

The author(s) declare that they have no competing interests.

## Authors' contributions

KOL carried out the research design, the fundraising, coordination of the organisational communication, the description of the technology, performed the interviews in the organisations, participated in the analysis of the organisations and the patients perspectives, and draft the manuscript. JO carried out the description of the health economic implications, and the analysis of the economy. EG participated in planning the interviews of the professional groups and the representatives of the management at the hospitals, and in analysis of the qualitative data from the organisations. FK participated in the preparation of the questionnaire to the patients, and in the data analysis related to the patients' perspective. MB participated in the preparation of the questionnaire to the patients, and in the data analysis related to the patients' perspective. All authors have read and approved the final manuscript.

## Pre-publication history

The pre-publication history for this paper can be accessed here:



## Figures and Tables

**Table 1:** Factors that promote or inhibit optimal nutritional carefrom the perspective of the production kitchen, the ward and the hospital management.

**Promotive factors**	**Inhibitory factors**
**The production kitchen's perspective:**	
Frequent contact to the nursing staff and patients	Lack of contact to the nursing staff and patients
That the kitchen follows the food right until it is served to the patient	That the kitchen does not follow or have any influence on how the food is served
High visibility within the organisation and hence the possibility to enhance knowledge of and respect for the kitchen staff's work from other professional groups	Low visibility within the organisation with the result that other professional groups know little about the kitchen staff's work.
	Lack of economic latitude
**The hospital ward's perspective:**	
That a professionally trained person works actively with nutritional care and has the necessary time to do so in practice	Lack of time for nutritional care, which is therefore accorded low priority
The presence of key personnel or committed individuals	That responsibility for the practical implementation of nutritional care is collective, but that only few are actively engaged
That the hospital ward can provide different types of food 24 hours a day, and that the staff serve these food items to the patients	That formal responsibility for nutritional care lies with the physicians, who rarely involve themselves in nutritional issues
The availability of cross-disciplinary guidelines for tracing patients at risk and for implementing nutritional care of various categories of patient	That the clinical dietician's expertise is underutilised
	That the nursing staff are unable to provide food outside the fixed mealtimes
**The hospital management's perspective**:	
Clear signals from management about the importance of optimal nutritional care	Weak or lacking signals from management about the importance of nutritional care
Access to management tools for quality assurance, support and assessment of nutritional care	Absence of management tools for obtaining the insight to be able to assess nutritional care at departmental level
Resources to follow-up and support implementation of nutritional care guidelines	

**Table 2:** Factors that promote or inhibit optimal nutritional care from the perspective of the patient.

**Promotive factors**	**Inhibitory factors**
That the patient has a real choice of menu at mealtimes	That the patient is not provided with the possibility to choose between menus adapted to preference, ability to chew, etc.
That the meal is adapted to the patient, for example with respect to consistency and size of portions	That the patient is not offered between-meal snacks and oral nutritional supplements for 12 of the 24 hours in a day
That the patient is informed about what the production kitchen can offer	That the patient is not informed about what the production kitchen can offer
Dialogue between the patient and nursing staff about the meals and menu choices	Lack of dialogue between the patient and nursing staff about individual needs and wishes
That presentation of the meals is appealing	That the patient's nutritional status is not monitored, and the patient's knowledge of his/her own weight loss is not utilized by the nursing staff

**Table 3:** Results of cost analysis for the three participating bed sections. 2003-price level.

**Variable**	Participating bed section at Hospital	Participating bed section at Hospital	Participating bed section at Hospital
	A *Department of cardiolog*	B, *Department of internal medicine*	C, *Department of geriatrics*
Number of patients hospitalised, total 2001/2002	992	1457	425
Number of patients aged 60+ hospitalised for more than 7 days	249	440	371
Number of undernourished patients if 35% are assumed to be undernourished during the admission	87	154	130
Number of saved bed days	296	524	441
Saved USD	66,412	117,355	98,952
Saved USD with full-time assistant dietician	26,181	36,893	18,490

**Table 4:** Number of admissions at Danish departments of internal medicine* in 2001. The patients were above the age of 59 years and hospitalised for at least 7 days.

Medical patients, variable	
Number of admissions	81,705
Average bed days (min = 7 days)	17.8 days
Average age (min. = 60 years)	76 years

*Departments of internal medicine include the speciality internal medicine and subspecialties like geriatrics, hepatology, haematology, infectious diseases, cardiology, medical allergology, endocrinology, gastroenterology, medico-pulmonary diseases, nephrology and rheumatology.

**Table 5:** Scenarios illustrating the cost saving potential for the entire country upon improvement of medical inpatients' nutritional care. The calculation only includes hospitalisation courses for patients above the age of 59 years who are hospitalised for at least 7 days (2003 – price level). Row II to VI presents the results of the sensitivity analysis. The variation in reduction of days (0.7 and 6.1 respectively) is taken from [14]. Extra allocation of resources (i.e. hiring an nutrition assistant) is included due to inspiration from [6]. The variation in the percentage of under-nourished is based on [5].

	Assumptions	Reduction in number of inpatient days	Annual cost reduction (USD)
I	35% under-nourished. 3.4 days' reduction in hospital stay. No allocation of extra resources.	97,229	22 m.
II	35% under-nourished. 0.7 days' reduction in hospital stay. No allocation of extra resources.	19,732	4 m.
III	35% under-nourished. 6.1 days' reduction in hospital stay. No allocation of extra resources.	175,012	39 m.
IV	35% under-nourished. 3.4 days' reduction in hospital stay. Extra allocation of resources (i.e. hiring of nutrition assistant).	97,229	6 m.
V	20% under-nourished. 3.4 days' reduction in hospital stay. No allocation of extra resources.	55,559	12 m.
VI	50% under-nourished. 3.4 days' reduction in hospital stay. No allocation of extra resources.	138,899	31 m.
